# Human descending aorta injury caused by brucellosis: A case report

**DOI:** 10.1097/MD.0000000000033764

**Published:** 2023-05-12

**Authors:** Xiao Li, Xiwei Sun, Yang Zhang, Sean X. Luo, Hang Yin, Hua Zhang, Zhongying Wang, Zhihua Cheng

**Affiliations:** a Department of Vascular Surgery, General Surgery Center, The First Hospital of Jilin University, Changchun, China.

**Keywords:** abdominal aortic pseudoaneurysm, brucellosis, digital subtraction angiography, infection, thoracic aortic ulcer

## Abstract

Brucellosis is one of the most common zoonotic diseases in the world. Although cardiovascular complications of human brucellosis account for only 3% of morbidity, they are the leading cause of death. Peripheral vascular disease due to brucellosis is rare and under-reported in the literature.

**Case presentation:**

Two patients with previous brucellosis, both of whom had been treated with anti-brucellosis, were admitted to vascular surgery for thoracic aortic ulcer and abdominal aortic pseudoaneurysm, respectively, with positive IgG antibody to brucellosis and negative IgM antibody to brucellosis, tube agglutination test, and blood culture. These 2 patients were successfully treated with aortic stent-graft implantation and followed up for 8 and 10 weeks without complications.

**Conclusions:**

Chronic damage to human blood vessels by brucellosis may not disappear with brucellosis treatment, and peripheral blood vessels should be examined annually in people previously diagnosed with brucellosis. Clinicians in related departments should pay attention to peripheral vascular complications of brucellosis.

## 1. Introduction

Brucellosis is a zoonotic infectious disease caused by Brucella, one of humans and animals’ most common infectious diseases.^[1]^ The disease can occur throughout the year, but it is more common in the abortion season of domestic animals, with a high incidence in late spring and early summer. Brucellosis is prevalent worldwide, with more than half a million new cases yearly, causing substantial economic losses.^[2]^ It is mainly found in Mediterranean countries, India, the Middle East, and Central and South America.^[3]^ In China, it is mainly prevalent in pastoral areas such as northwest, northeast, Inner Mongolia, and Qinghai-Tibet Plateau.^[3]^ Brucellosis is an occupational disease that is commonly seen in occupations such as veterinarians, animal husbandry workers, slaughter workers, milking workers, and fur workers.^[4]^ Animals carrying Brucella and diseased animals were the source of infection. Brucellosis can be transmitted to humans through skin and mucosa, the digestive tract, the respiratory tract, and other transmission routes.^[5]^ The main manifestations of acute brucellosis infection are fever, fatigue, hyperhidrosis, and muscle and joint soreness, and a few patients may have heart, kidney, and nervous system involvement.^[6]^ The manifestations of chronic Brucella infection are mainly organic damage, such as nerve damage (such as peripheral neuritis), genitourinary system damage (such as orchitis), cardiovascular system damage (such as endocarditis), musculoskeletal system damage (such as sizeable joint damage) and so on.^[6]^ Brucellosis is one of the most mimetic infectious diseases in the world. It is like the clinical manifestations of various systemic diseases, which significantly interfere with the diagnosis of clinical workers, often leading to misdiagnosis and delayed treatment.^[7]^ Endocarditis (3% of cases) is the most severe complication, of which the aortic valve is the most affected structure, and multiple valve involvement is also common in such patients, accounting for most of the 5% overall mortality from human brucellosis.^[8]^ Although brucellosis affects multiple systems, vascular complications associated with brucellosis infection, both arterial and venous, have rarely been reported.

This report presents 2 patients with a history of brucellosis, positive IgG antibody, negative IgM antibody, negative tube agglutination test for Brucella, and negative blood culture, who were admitted to vascular surgery for thoracic aortic ulcer and abdominal aortic pseudoaneurysm, respectively. Most studies investigating brucellosis involving peripheral arteries have been case reports and small case series. Most of these cases are peripheral vascular complications of brucellosis during acute infection. No peripheral vascular complications have been reported in patients with previous brucellosis after their recovery from anti-brucellosis therapy. More information is needed on treatments and cases of this disease.

## 2. Case presentation

### 2.1. Case 1

**Figure 1.**
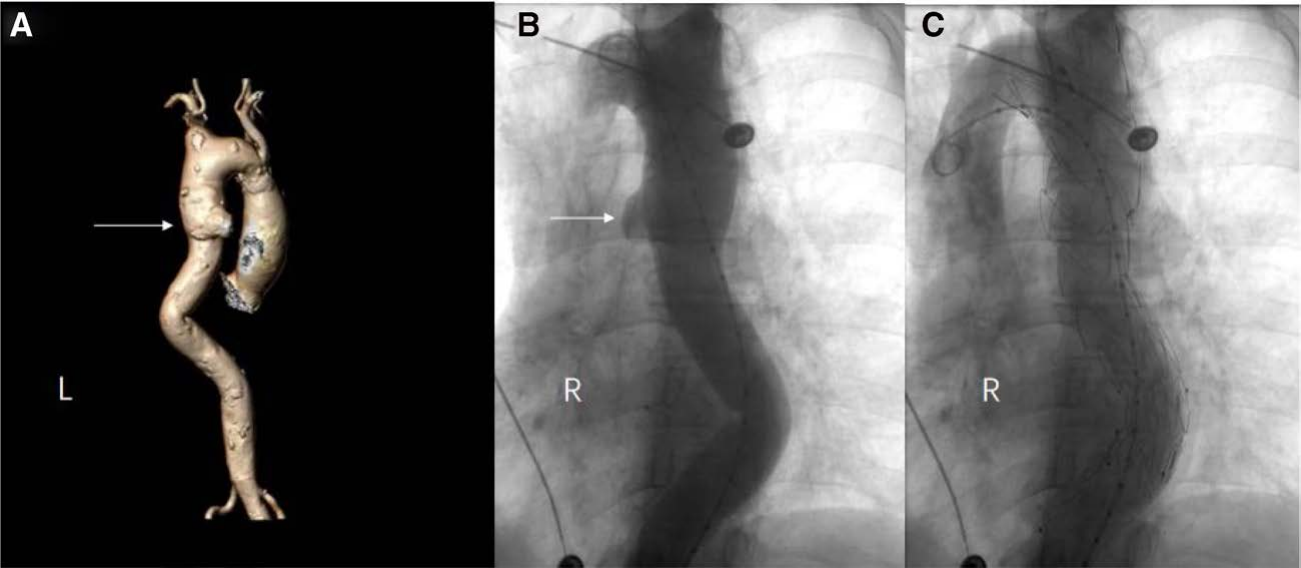
(A) CTA of the thoracoabdominal aorta shows a penetrating ulcer in thoracic aorta. (B) Intraoperative DSA shows an aortic ulcer approximately 2 cm distal to the left subclavian artery on the lesser curvature of the aorta. (C) Intraoperative DSA shows no endoleaks. CTA = computed tomography angiography, DSA = digital subtraction angiography.

A 65-year-old China woman was admitted to the First Hospital of Jilin University due to sudden chest pain for 3 days. The patient had no previous history of surgery, trauma, hypertension, diabetes, coronary heart disease, tuberculosis, syphilis, infectious mononucleosis, human immunodeficiency virus, and hepatitis B infection. The patient’s body temperature, blood pressure, heart rate, and respiration were average on admission, and physical examination showed no other abnormalities. The patient underwent multi-slice spiral CT angiography of the thoracoabdominal aorta, which showed penetrating ulceration of the thoracic aorta (Fig. 1A). Electrocardiogram and cardiac ultrasound were normal. Laboratory tests showed pro-B-type natriuretic peptide (PRO-LPBN) 145.0 pg/mL (normal range 0–125 pg/mL), and other laboratory tests showed no obvious abnormality. The patient complained that he had raised goats for 14 years and was diagnosed with brucellosis 8 years ago and was treated with doxycycline and rifampicin for 6 weeks against brucellosis. On admission, the patient was found to be positive for Brucella IgG antibody and negative for Brucella IgM antibody, brucellosis tube agglutination test, and blood culture. We performed endovascular thoracic aortic exclusion on the premise that the patient and his family knew the patient’s condition and agreed to surgical treatment. A 6F sheath (Terumo Corporation, Japan) was retrogradely inserted into the right common femoral artery intraoperatively. A digital subtraction angiography using a pigtail catheter revealed an aortic ulcer approximately 2 cm distal to the left subclavian artery on the lesser curvature of the aorta (Fig. 1B). The left vertebral artery was well developed. Lunderquist® Extra-Stiff Wire Guide (Cook Medical Inc., Denmark) tip was introduced into the descending aorta, and a 24 mm × 24 mm × 82 mm ENDURANT-covered aortic stent (Medtronic, Minneapolis, MN) and a 38 mm × 38 mm × 150 mm ENDURANT-covered aortic stent (Medtronic) were introduced with the aid of a guidewire. The aortic stent was released entirely at the medial edge of the origin of the left subclavian artery, and the distal end entered the descending aorta smoothly. At the final angiogram, no intravascular leak was noted. The bilateral common carotid artery and left subclavian artery were well-developed without leakage (Fig. 1C). The surgery was a success. The patient’s vital signs remained stable throughout the procedure. After surgery, the patient did not receive anti-brucella treatment under the comprehensive evaluation of the infectious doctor. The patient was followed up for 8 weeks after surgery and had no notable discomfort symptoms.

### 2.2. Case 2

**Figure 2.**
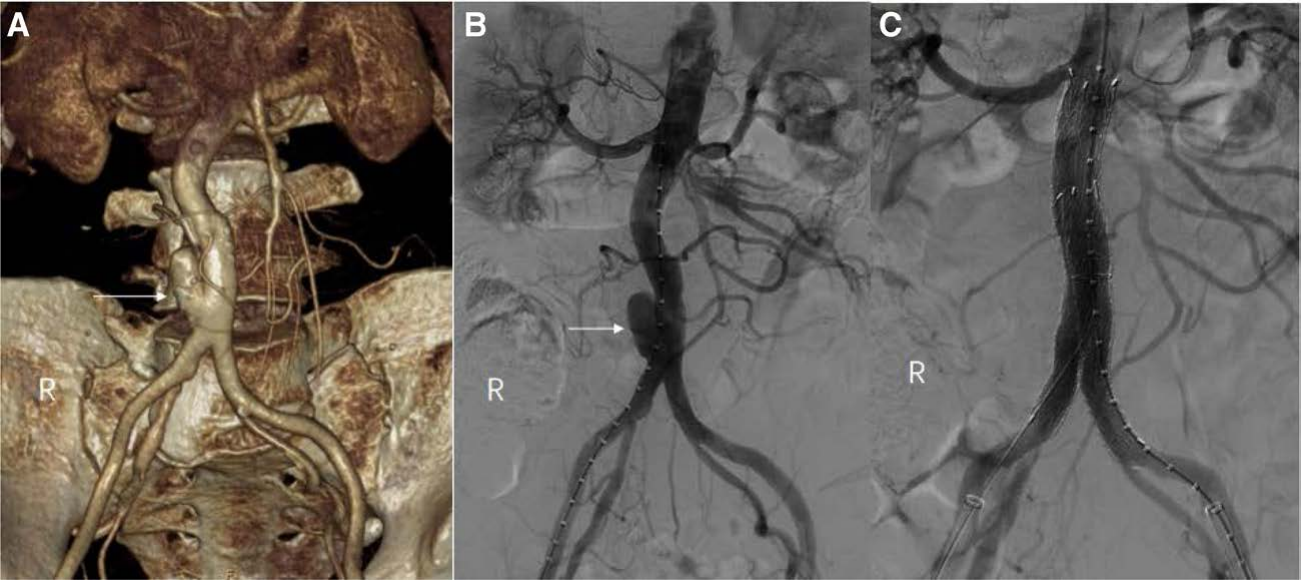
(A) Abdominal aorta CTA shows pseudoaneurysm formation in the lower abdominal aorta. (B) Intraoperative DSA shows a pseudoaneurysm at the end of the abdominal aorta. (C) DSA showed no endoleaks. CTA = computed tomography angiography, DSA = digital subtraction angiography.

A 64-year-old China man was admitted to the First Hospital of Jilin University due to sudden low back pain for 7 days. The patient underwent cholecystectomy 30 years ago for cholecystitis. The patient had been working in goat breeding for 10 years and was diagnosed with brucellosis 5 years ago and treated with gentamicin for 4 weeks and rifampicin and doxycycline for 12 weeks. The patient’s wife and son also had brucellosis. The patient had no hypertension, diabetes, coronary heart disease, tuberculosis, syphilis, infectious mononucleosis, human immunodeficiency virus, and hepatitis B infection. No recent history of trauma. Physical examination on admission showed apparent deep abdominal tenderness, no abdominal muscle tension, rebound tenderness, traditional abdominal percussion, average body temperature, blood pressure, heart rate, and respiration. On admission, the patient was found to be positive for Brucella IgG antibody and negative for brucellosis IgM antibody, brucellosis tube agglutination test, and blood culture. White blood cell count, C-reactive protein, and erythrocyte sedimentation rate were within normal range. CT angiography of the abdominal aorta showed pseudoaneurysm formation in the lower abdominal aorta, with a maximum lumen diameter of about 3.5 cm, about 5.4 cm below the renal artery, and slightly low-density shadow wrapping in the peripheral part (Fig. 2A). Electrocardiograph and cardiac ultrasound showed no abnormality. Laboratory tests showed no significant abnormalities. Endovascular exclusion of abdominal aortic pseudoaneurysm was performed when the patient and his family understood the patient’s condition and agreed to surgical treatment.

A TAG-covered stent graft (W. L. Gore & Associates, Inc., Flagstaff, AE) isolated the pseudoaneurysm. A 6F sheath (Terumo Corporation) was retrogradely inserted into the right common femoral artery intraoperatively. A pseudoaneurysm was identified on digital subtraction angiography with a pigtail catheter (Fig. 2B). Then, with the help of a Lunderquist® Extra-Stiff Wire Guide (Cook Medical Inc.), a 22F sheath (W. L. Gore & Associates, Inc.) was introduced, and a 23 mm × 12 mm × 120 mm TAG-coated stent-graft and a 12 mm × 10 mm × 100 mm endoprosthesis contralateral leg (W.L. Gore & Associates, Inc.) were implanted to isolate the pseudoaneurysm. In the final angiography, no endoleaks were identified (Fig. 2C). The surgery was a success. The patient’s vital signs remained stable throughout the procedure. After surgery, the patient did not receive anti-brucella treatment under the comprehensive evaluation of the infectious doctor. The patient was followed up for 10 weeks after surgery and had no notable discomfort symptoms.

## 3. Discussion and conclusions

Brucellosis is a disease with diverse manifestations that can affect multiple systems of the human body. Brucellosis may remain undetected for long periods before clinical manifestations become apparent. Although brucellosis can lead to endarteritis, which can lead to a series of vascular complications, brucellosis vascular complications are rare. Although brucellosis rarely involves peripheral blood vessels, it will seriously endanger the life of patients once it occurs. In a review published in 2022, Siem et al reported that aortoiliac artery disease involving brucellosis is an underdiagnosed and underreported disease.^[9]^ Brucellosis involving large arteries has occult and nonspecific manifestations and requires high suspicion. Any acute chest or abdominal pain should be suspected of aortic involvement, and a routine aortic examination should be performed to confirm aortic involvement. Imaging is an important diagnostic method for the vascular manifestations of aortoiliac complications of brucellosis. Ultrasonography as the initial imaging modality for diagnosing the infectious peripheral vascular disease is unreliable, and multi-slice spiral CT angiography is currently the imaging modality of preferred choice for evaluating suspected infectious arterial disease.^[10]^ Vascular surgeons may not be familiar with or ignore brucellosis, but they are also the leading doctors dealing with peripheral vascular diseases. Therefore, many patients do not receive timely diagnosis and treatment after this adverse event, which increases the risk of death from this disease. Therefore, improving the understanding of peripheral vascular complications caused by brucellosis among doctors in relevant departments is very important.

Brucella can infect vascular endothelial cells and live inside the cells, so it is an intracellular parasite. Bacteria and toxins play a significant role in the acute phase, whereas delayed anaphylaxis and granuloma formation play significant roles in the chronic phase. Immunocompromised patients, such as those with diabetes, alcohol abuse, a high-fat diet, or chemotherapy, are at increased risk for vascular complications when brucellosis is combined. Age greater than 50 years is considered a risk factor because of the higher incidence of arteriosclerosis, and the irregular surface of the intima of the blood vessels becomes the site of brucellosis attachment.^[11,12]^ The 2 reported cases, previously diagnosed with brucellosis, were not in the acute infection stage when they were admitted to the hospital, and they also presented with peripheral vascular complications. This indicates that vascular damage caused by brucellosis does not disappear with the treatment of brucellosis, and vascular damage manifests with increasing age. This does not rule out a combination of brucellosis damage to blood vessels and degeneration of the vessels. Therefore, patients with a history of brucellosis should increase the annual physical examination of peripheral arteries; timely detection of peripheral vascular lesions, early treatment, and prevention of disease progression is more beneficial to patients.

Current guidelines suggest a triple combination antibiotic regimen for Brucella endocarditis, including an aminoglycoside for 4 weeks (gentamicin 5 mg/kg/d) plus rifampicin (600 e 900 mg orally once daily) and doxycycline (100 mg orally twice daily) both for at least 12 weeks.^[9,13]^ Monotherapy regimens and duration shorter than 6 weeks are not accepted treatment strategies for brucellosis, causing high relapse rates and complications.^[9,13–15]^ In the process of anti-brucellosis treatment, the side effects caused by these antibiotics cannot be ignored. Chinese herbal medicine is used for anti-brucellosis treatment in some areas of China. This treatment regimen has not been reported in the literature.

Traditionally, aneurysm resection, local debridement, and graft replacement have been used to treat this disease.^[16]^ The traditional surgical approach makes a huge incision in the human body and replaces the affected blood vessel with an artificial blood vessel. The patient’s surgical trauma is enormous, there is a high risk of rupture during the operation, and the patient has a prolonged postoperative recovery time. With the appearance and development of endovascular repair, it has been favored by more and more clinical workers because of its advantages such as less trauma, low incidence of complications, rapid postoperative recovery, avoidance of extracorporeal circulation, and massive blood transfusion.^[9,17–20]^ However, endovascular repair technology has strict standards for stent placement and sealing, and it is difficult to obtain the damaged vascular tissue for pathological analysis. Many still dispute whether this technology can be used for this infectious lesion. Both of our patients were treated with an endovascular repair technique and were followed up for 8 and 10 weeks, respectively, after surgery. The patients survived well without relevant complications. We will conduct further follow-ups.

In conclusion, peripheral vascular involvement of brucellosis is an underdiagnosed and under-reported disease that should be brought to the attention of physicians in relevant departments. Chronic damage to human blood vessels by brucellosis may not disappear with brucellosis treatment, and annual peripheral vascular examinations should be increased in people previously diagnosed with brucellosis. Although endovascular repair therapy has become a safe and successful option for the treatment of peripheral vascular complications of brucellosis, there still needs to be more experience in this area. For individuals with a history of contact with cattle and sheep, long-term fever of unknown origin, back and abdominal pain, and macrovascular disease caused by Brucella infection should be considered during differential diagnosis, and blood culture, antibody detection, computed tomography angiography, and other relevant examinations should be carried out in time to avoid life-threatening delay in treatment.

## Author contributions

**Conceptualization:** Xiao Li, Zhihua Cheng.

Data curation: Xiao Li.

Formal analysis: Xiao Li, Xiwei Sun, Yang Zhang, Zhihua Cheng.

Investigation: Xiao Li, Zhihua Cheng.

Methodology: Xiao Li, Zhihua Cheng.

Software: Xiao Li, Xiwei Sun, Zhihua Cheng.

Supervision: Xiwei Sun, Yang Zhang, Sean X. Luo, Hang Yin, Hua Zhang, Zhongying Wang, Zhihua Cheng.

Writing – original draft: Xiao Li.

Writing – review & editing: Zhihua Cheng.
